# Reviewed article: Santos TSS, Guimarães LMF, Freitas PP, Campos SF, Cardoso CS, Toral N, Lopes ACS. Percurso metodológico qualitativo para elaboração de Instrutivo de Manejo da Obesidade no SUS, 2018-2020. Epidemiol Serv Saude. 2025:34;e20240365

**DOI:** 10.1590/S2237-96222025v34e20240365.a

**Published:** 2025-04-14

**Authors:** Haroldo da Silva Ferreira

**Affiliations:** Universidade Federal de Alagoas

**Table:** 

Rating	Excellent	Good	Average	Below average	Poor
Interest		✓			
Quality			✓		
Originality	✓				
Overall		✓			

**Table:** 

Questionnaire	Yes	No	Not applicable
Does the manuscript contain new and significant information to justify publication?	✓		
Does the Abstract (Summary) clearly and accurately describe the content of the article?	✓		
Is the problem significant and concisely stated?	✓		
Are the methods described comprehensively?		✓	
Are the interpretations and conclusions justified by the results?		✓	
Is adequate reference made to other work in the field?	✓		

## Manuscript Structure:

Length of article is: Too short

Number of tables is: Adequate

Number of figures is: Adequate

Please state any conflict(s) of interest that you have in relation to the review of this paper (state “none” if this is not applicable): None

